# Monitoring of Ammonia in Biomass Combustion Flue Gas Using a Zeolite-Based Capacitive Sensor

**DOI:** 10.3390/s25175519

**Published:** 2025-09-04

**Authors:** Thomas Wöhrl, Mario König, Ralf Moos, Gunter Hagen

**Affiliations:** 1 Department of Functional Materials, Zentrum für Energietechnik (ZET), University of Bayreuth, D-95440 Bayreuth, Germany; 2 Deutsches Biomasseforschungszentrum Gemeinnützige GmbH (DBFZ), D-04347 Leipzig, Germany

**Keywords:** ammonia detection, biomass combustion, flue gas aftertreatment, zeolite-based gas sensor

## Abstract

The emissions from biomass combustion systems have recently been the subject of increased attention. In addition to elevated concentrations of particulate matter and hydrocarbons (HCs) in the flue gas, significant levels of NO_x_ emissions occur depending on the used fuel, such as biogenic residues. In response to legal requirements, owners of medium-sized plants (≈100 kW) are now also forced to minimize these emissions by means of selective catalytic reduction systems (SCR). The implementation of a selective sensor is essential for the efficient dosing of the reducing agent, which is converted to ammonia (NH_3_) in the flue gas. Preliminary laboratory investigations on a capacitive NH_3_ sensor based on a zeolite functional film have demonstrated a high sensitivity to ammonia with minimal cross-influences from H_2_O and NO_x_. Further investigations concern the application of this sensor in the real flue gas of an ordinary wood-burning stove and of combustion plants for biogenic residues with an ammonia dosage. The findings demonstrate a high degree of agreement between the NH_3_ concentration measured by the sensor and an FTIR spectrometer. Furthermore, the investigation of the long-term stability of the sensor and the poisoning effects of SO_2_ and HCl are of particular relevance to the laboratory measurements in this study, which show promising results.

## 1. Introduction

Ammonia (NH_3_) is a crucial component in the selective catalytic reduction (SCR) of nitrogen oxides (NO_x_), which are produced during combustion processes. While SCR technology is well established in the transport sector, recent regulatory developments have intensified its relevance for stationary biomass combustion systems [1,2]. In contrast to fossil fuel combustion, where NO_x_ formation predominantly results from thermal and prompt mechanisms involving atmospheric nitrogen, biomass combustion exhibits a significantly higher contribution from fuel-bound nitrogen compounds [3,4,5]. These compounds, bound during the growth phase of the biomass, are released as NO_x_ during combustion, thereby contributing to atmospheric pollution.

The environmental and health impacts of NO_x_ emissions have been extensively documented. NO_x_ has been identified as a significant contributing factor to the formation of ground-level ozone and particulate matter, both of which present potential health risks [6,7]. In addition, nitrogen oxide contributes to the formation of acid rain, thereby indirectly accelerating climate change and environmental degradation [8]. In order to reduce NO_x_ emissions, ammonia (NH_3_) is added to the flue gas of combustion plants. In a special catalytic converter, NO_x_ gets converted into harmless nitrogen and water vapor. The following Equations (1) and (2) describe the so-called SCR reactions (with SCR denoting selective catalytic reduction) [9]:

4NH_3_ + 4NO + O_2_ → 4N_2_ + 6H_2_O (1)

4NH_3_ + 2NO + 2NO_2_ → 4N_2_ + 6H_2_O (2)

In practical applications, ammonia is added to the exhaust gas as a 32.5% aqueous urea solution (AdBlue^TM^) and reacts with NO_x_, with optimal conversion rates at operating temperatures between 250 and 450 °C [10,11,12]. Deviations from this range can lead to incomplete urea decomposition at lower temperatures or the formation of undesirable byproducts such as nitrous oxide (N_2_O) at elevated temperatures [2,13].

With the introduction of the 44th BImSchV (Ordinance on the Implementation of the Federal Immission Control Act, Germany), the emission limits for medium-sized combustion plants in Germany have been significantly tightened [14]. Since 2019, output-related NO_x_ limit values for new plants between 200 and 750 mg/Nm^3^ (referred to as 6 vol.% O_2_) have been in force, which generally cannot be met without SCR technology [15]. At the same time, an ammonia slip limit of 30 mg/Nm^3^ was introduced. This can only be achieved with optimal dosing and control of the urea injection system [15,16].

The integration of SCR technology into biomass combustion systems presents specific challenges. High concentrations of particulate matter and soot can accumulate on the catalyst surface, potentially decreasing its effectiveness. Additionally, toxic byproducts of combustion, such as phosphorus, sulfur, and chlorine compounds, may lead to an irreversible deactivation of the catalyst material. In response to this issue, research has been focused on the development of robust catalyst formulations. These include vanadium-based catalysts, which, in combination with various metal oxides such as MnO_x_, NiO_x_, or CuO_x_, exhibit high activity with low poisoning characteristics [17,18]. As an alternative, catalysts consisting of zeolites loaded with metal or noble metal species also offer enhanced thermal stability [2,17]. The integration of SCR catalysts with simultaneous filtration of flue gas particles is a matter of current interest, as it enables the implementation of cost-effective aftermarket solutions for combustion plants [1,19].

Beyond emission abatement, continuous monitoring of the flue gas composition is essential for effective exhaust gas aftertreatment control. The combustion of biomass is marked by dynamic operating conditions, including fluctuations in temperature and variable fuel compositions. This necessitates the implementation of adaptive dosing strategies for the reducing agent [20,21]. Accurate control of NH_3_ injection is critical, as both under- and overdosing may result in the NO_x_ limit value being exceeded or in an undesirable ammonia slip [22].

Emission monitoring currently varies depending on the power output of the combustion plant. NO_x_ sensors derived from automotive applications are commonly used for continuous measurement, but their simultaneous cross-sensitivity to NH_3_ limits the ability to make specific statements about the flue gas composition [14,23,24]. Optical systems such as Fourier-transform infrared (FTIR) spectrometers offer higher accuracy. However, these systems are not cost-effective due to their high acquisition and maintenance costs [25,26]. Therefore, continuous measurement is not required for many plants in the lower power range [27]. Consequently, many low-power plants rely on periodic flue gas analyses, e.g., conducted annually, which may result in inaccurate dosing of the reducing agent and the associated increase in pollutant emissions.

To address these limitations, the development and implementation of selective ammonia sensors is essential. These sensors enable continuous and selective measurement of the ammonia content in the flue gas, thereby providing the signal for adaptive control of the reducing agent supply in the future.

Various sensor technologies have been developed to meet the demanding requirements of these environments. These include solid-state electrolyte sensors, metal-oxide semiconductor (MOS) sensors, and optical detection systems [28].

Solid-state sensors, especially mixed-potential types, operate based on electrochemical reactions at the interface of a solid electrolyte and electrodes. These reactions generate a voltage signal that correlates with the ammonia concentration [29]. Their key advantages include high thermal stability (up to 700 °C), long-term durability, and low power consumption [30]. However, they often demonstrate limited selectivity, and their performance can degrade over time due to material aging [31,32,33].

MOS sensors detect ammonia through changes in the electrical resistance of metal-oxide materials such as (mostly doped) SnO_2_, ZnO, or WO_3_ [34]. These sensors are valued for their high sensitivity, fast response, and relatively low cost. Recent advances in nanostructuring and doping have improved their performance significantly. Nevertheless, elevated operating temperatures (200–400 °C) are typically required, which may result in signal drift over time due to ageing of the sensitive material [35]. Additionally, cross-sensitivity with other gases is of major concern [36].

Optical sensors, including UV/Vis absorption, non-dispersive infrared (NDIR), and photoacoustic techniques, rely on the interaction of ammonia molecules with light. These sensors offer excellent selectivity and can operate without direct contact with the gas stream, making them ideal for real-time monitoring. Their drawbacks include high cost, sensitivity to environmental factors such as dust and humidity, and the need for regular calibration and maintenance [37].

The development of advanced sensing technologies, including photoacoustic sensors, surface acoustic wave (SAW) devices, and field-effect transistor sensors, has also led to significant progress in the field of ammonia detection [35,38]. These systems offer promising sensitivity and miniaturization potential, but often face challenges in robustness and integration into commercial settings [26].

The development of a capacitive ammonia sensor with a zeolite-based functional film on a ceramic substrate introduces a new sensing concept designed for use in high-temperature and chemically aggressive environments [39]. Zeolites offer high thermal stability and molecular selectivity, making them ideal for the adsorption of ammonia in complex gas mixtures [40]. Integrating the zeolite film into a capacitive sensing architecture enables detection via changes in dielectric properties, allowing for low-power operation. Using a ceramic substrate also enhances the sensor’s mechanical and thermal robustness, ensuring stability in harsh environments. In the framework of this study, this sensor principle should be specially tested for use in the flue gas of biomass plants.

## 2. Materials and Methods

This section explains the fundamentals of the sensor and the various experimental setups. It includes an introduction to the sensor element and a description of the test benches used in laboratory and in flue gas environments.

### 2.1. Sensor Setup

The sensor is composed of a planar structure, and its fabrication primarily utilizes screen printing processes, thereby ensuring cost-effective production. A detailed overview of the manufacturing process of the sensor is given by [41,42].

The essential component of the sensor is the zeolite functional film, which is applied on an alumina substrate. The electrical properties of the zeolite film are measured using an interdigital electrode structure (IDE consisting of gold with 20 μm line width and spacing), located between the substrate and the zeolite film [43,44]. Zeolites are aluminosilicates that are known for their ring-shaped framework structure and the high surface area for the adsorption of various gas species [45,46,47]. The ZSM5 zeolite used here (ALSI-Penta GmbH, Schwandorf, Germany) features an MFI (metal framework inverted) structure. In recent years, it has been applied as a catalyst material for SCR (selective catalytic reduction) systems [48,49,50]. The notable capacity of this zeolite type to adsorb significant quantities of ammonia makes it a suitable material for use as a functional sensor film [51,52]. In the proton-exchanged variant selected here, it is expected that catalytic activity will be as low as possible, as potential conversion reactions on the sensor surface at an operating temperature of *T*_sensor_ = 400 °C could lead to incorrect measurement results [42,53]. In earlier investigations, zeolites with a Si/Al ratio of 27 showed an optimum balance in terms of cross-sensitivity to H_2_O and NO_x_ [42]. The zeolite film that was applied in this study by screen-printing exhibited a thickness of approximately 35 µm (after firing). This film thickness was found to generate the most significant NH_3_ response in our investigations.

**Figure 1:**
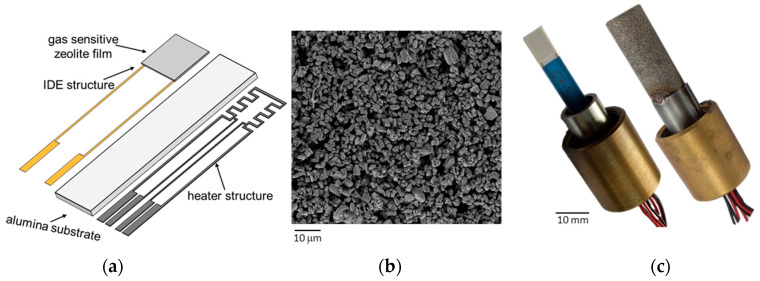
(**a**) Schematic structure of the screen-printed sensor; (**b**) SEM image of the surface of the porous zeolite film; (**c**) protective cap variants for use in laboratory (left, no cap) and real flue gas (right) measurements.

The sensor is heated via a four-wire platinum heater structure on the reverse side. This configuration enables precise temperature regulation across the functional film, even in conditions of variable flow and temperature of the flue gas [54]. The structure of the sensor and an SEM image of the surface of the porous zeolite film are shown in Figure 1a,b.

The capacitance of the sensor is the measurand, which is used for determining the NH_3_ concentration. For this purpose, a sinusoidal voltage with a frequency of 700 kHz and an effective voltage of 250 mV is applied to the interdigital electrodes (IDEs). The capacitance value of the sensor was determined by evaluating the resulting current and the phase shift [55,56].

A universally compatible clamp connection was used for installing the sensors for characterization in the laboratory and in real flue gas. Different protective cap variants can also be tested with this kind of connector [57]. For measurements in the laboratory with lower volume flows, an open protective cap was used. With this, the sensor tip extends into the synthetic exhaust gas without additional protection (see Figure 1c, left). A protective cap equipped with a sinter-metal filter with a pore size of 50 μm was used for the later measurements in the flue gas (see Figure 1c, right). This type of protective cap is designed to prevent the entry of larger soot or ash particles that could have a negative effect on the sensor. The use of these protective cap types is based on experience gained from previous sensor developments. In these cases, sintered metal protective caps demonstrated a high filtering effect against particles without affecting the sensor response to the target gas.

### 2.2. Experimental Setup in the Laboratory

**Figure A1:**
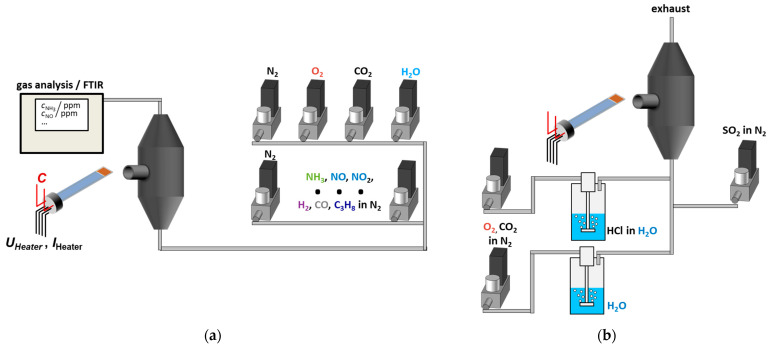
Measurement setups in the laboratory. (**a**) Sensor test bench for characterization of the sensors. (**b**) Measuring chamber and dosing system of SO_2_ and HCl for sensor poisoning.

A gas mixing system for synthetic exhaust gases was available in the lab for sensor characterization. The configuration of the system is illustrated in Appendix A.1 Figure A1a and consists of two lines. One line is used for dosing the base gases, which represent the major components of the exhaust gas. This includes nitrogen (N_2_), oxygen (O_2_), carbon dioxide (CO_2_), and water vapor (H_2_O). With the second line, gases with lower concentrations (in the ppm range) are dosed, such as NH_3_, NO_x_, C_3_H_8_, CO, and H_2_. These gases are then added to the main flow downstream humidification. The total volume flow rate during the measurement on this system was 6 L/min. The composition of the dosed gas was analyzed after the sensor measuring chamber with an FTIR spectrometer (MKS MultiGas 2030 FTIR Analyzer, MKS Inc., Andover (MA), USA), as far as possible for the dosed gases.

A second measurement setup was used for poisoning tests (see Appendix A.1 Figure A1b), as the dosing of potentially aggressive gas components could damage the piping of the sensor test bench. This setup, therefore, contained fewer mass flow controllers (MFCs) for gas dosing. Accordingly, pre- and post-characterization of the sensors was carried out to determine the ammonia response, in each case on the sensor test bench described above. Synthetic exhaust gas from a gas bottle with 3 vol.% CO_2_ and 10 vol.% O_2_ in N_2_ was used as the base gas for the poisoning measurements. The water dosage was carried out by passing the gas flow through a wash bottle filled with water, whereby a water content of approx. 2 vol.% was achieved. For the poisoning tests, SO_2_ (3 vol.% in N_2_) could optionally be dosed via an additional MFC, or part of the base gas could be passed through a hydrochloric acid solution (10 vol.% HCl). The total volume flow was set at 3 L/min for each of these measurements. Due to dilution with the base gas, concentrations of about 200 ppm SO_2_ and 300 ppm HCl were dosed. The dosage of lower concentrations was not possible because of the low volume flows that exceeded the lower mass flow limits of the mass flow controllers.

### 2.3. Experimental Setup for Measurements in Flue Gas

**Figure A2:**
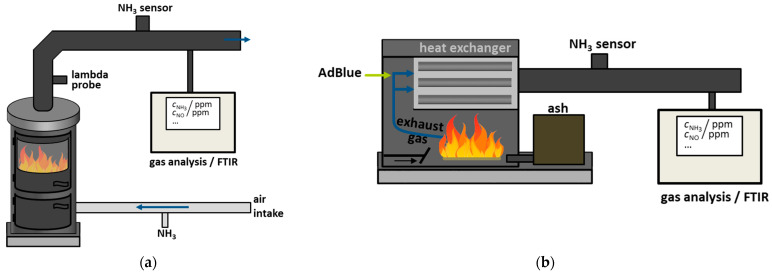
Schematic setup for investigations in real flue gas of biomass combustion systems (**a**) on a wood-burning furnace for residential use and (**b**) on multi-fuel boilers with straw pellets for hot water supply.

A variety of combustion systems were available for the purpose of conducting real flue gas measurements. The initial tests were conducted using a standard wood-burning stove (model LEDA Unica, Leer, Germany, provided by the Deutsches Biomasseforschungszentrum, DBFZ, Leipzig, Germany) with a heat output of 5 kW. The primary function of the stove was as a flue gas supplier, and it was operated at nominal load using spruce logs. The manual operation of the stove, for instance, through the addition of firewood and the manual regulation of the air supply to the combustion chamber, resulted in variable conditions for the composition of the flue gas and the temperature. Ammonia was dosed using an NH_3_/H_2_O mixture (25 wt.% NH_3_), through which a certain flow of nitrogen was passed and saturated. This gas flow was then mixed with the intake air of the furnace. The flow rate of the NH_3_ gas mixture was regulated by a needle valve, thereby affecting the NH_3_ concentration in the flue gas. The resulting gas emissions were also determined in this experiment using the FTIR spectrometer mentioned above. A schematic representation of the experimental setup can be found in Appendix A.1 in Figure A2a.

In contrast, two combustion plants at the Deutsches Biomasseforschungszentrum (DBFZ) were used, whose fuel and air supply were controlled automatically. They offered fewer dynamic conditions. Additionally, an ash removal system is integrated into the firing systems, thereby protecting the boiler from the issues of slagging and inefficient combustion of the biomass. Depending on the operating mode, this can lead to a significant increase in emissions, such as CO and NO_x_, for a certain period of time. The two multi-fuel boilers were operated with straw pellets and demonstrate an output range between 120 kW (REKA with moving grate) and 49 kW (Ökotherm Compact C0 with ash slider). AdBlue^TM^ was dosed into the flue gas with the help of a nozzle, which was placed around the heat exchanger used for hot water generation. This was necessary because temperatures of over 250 °C are required for AdBlue^TM^ conversion. The ammonia sensor was positioned within the flue gas pipe, with a downstream measuring probe that led to an FTIR spectrometer (Ansyco, Meyerton, South Africa). The scheme of the setup can be seen in Figure A2b in Appendix A.1.

## 3. Results

The sensors were pre-characterized in the laboratory setup previously described to determine the fundamental behavior of the sensor. This included sensitivity measurements relating to the target gas NH_3_, as well as cross-sensitivity measurements with gas components that occur to a higher extent in the flue gas of biomass combustion systems. Some components may have the potential to result in a shift in sensor signals in combination with NH_3_. Moreover, investigations were conducted to address sensor poisoning with SO_2_ and HCl. The sensor’s long-term stability was also examined.

### 3.1. Ammonia Sensitivity and Cross-Sensitivities

**Figure 2:**
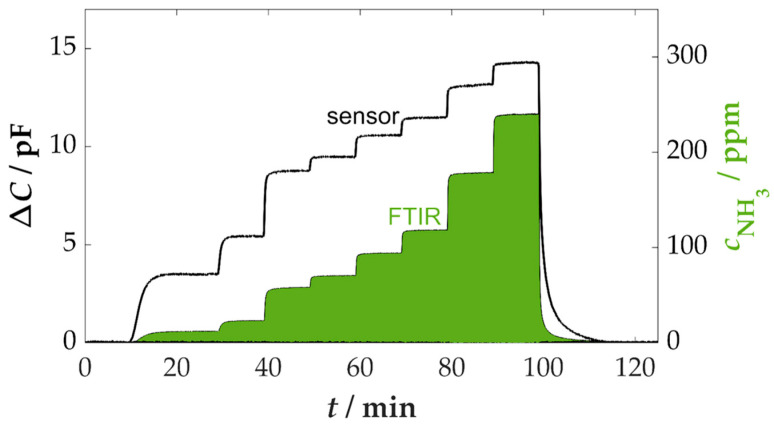
Time course of the capacitance change of the sensor with increasing ammonia concentration (0–240 ppm) in the synthetic exhaust gas; base gas: 5 vol.% H_2_O, 10 vol.% O_2_, 3 vol.% CO_2_ in N_2_, *T*_sensor_ = 400 °C.

The NH_3_ sensitivity was determined up to a maximum concentration of approximately 240 ppm, which is within the range of NO_x_ concentrations that occur, although they can vary depending on the combustible materials [21,58]. The fundamental sensor behavior is illustrated in Figure 2, which presents the time course of a measurement sequence.

The evaluation of the sensor result is shown as a change in capacitance Δ*C*, which is the difference between the measured capacitance and the base capacitance determined in the base gas without the presence of NH_3_ (an average value of 58.2 pF between 0 and 5 min in this measurement). The base capacitance of the sensor consists of several components. This includes not only the functional film, but also parts of the sensor substrate (≈40–45 pF) and feed lines on the substrate and by cables (≈1–2 pF) [43,59,60]. These influences can vary slightly between different sensors and can depend on the positioning of the measuring lines. For later use in real flue gas, a single calibration is therefore necessary by determining the base capacitance without AdBlue^TM^ dosing.

**Figure 3:**
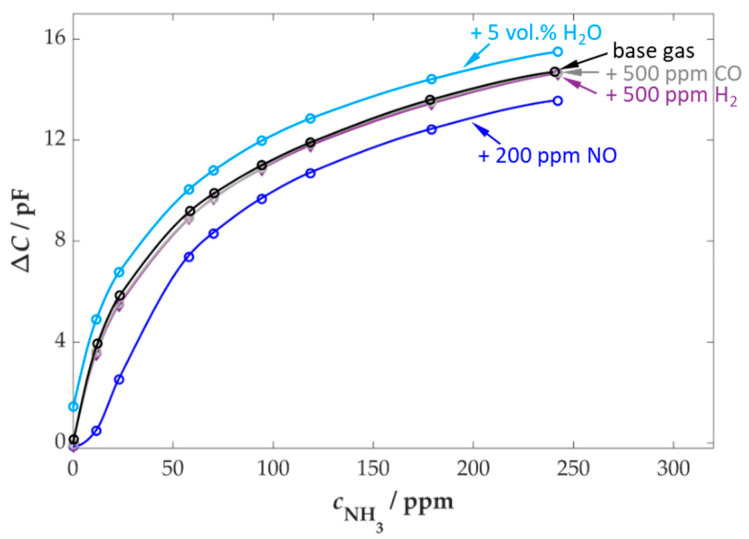
Characteristic curves of the sensor as a function of the NH_3_ concentration with additional variation of the gas components for cross-sensitivity testing; base gas: 5 vol.% H_2_O, 10 vol.% O_2_, 3 vol.% CO_2_ in N_2_, *T*_sensor_ = 400 °C.

The change in capacitance over time exhibited a quick response to variations in the NH_3_ concentration. A return to the initial value was also observed when the NH_3_ dosing was switched off. A response time is not very meaningful due to the adsorption and desorption processes of ammonia in the piping to the FTIR spectrometer, which lead to time delays in the determination of the concentration [61,62]. The further evaluation of the data was based on the calculation of characteristic curves for the steady-state values of the sensor and FTIR data. In Figure 3, the characteristic curve of the measurement in Figure 2 is presented. Thereafter, the influence of varying gas components on the NH_3_ sensitivity of the sensor was investigated in subsequent phases of the measurement. In order to further investigate the effects of additional gas components, such as CO and NO_x_, as well as the effects of varying base gas components, such as H_2_O, the measurement curve from Figure 2 was repeated on several occasions. A selection of the cross-sensitivities that were investigated is also demonstrated in Figure 3.

The sensor’s fundamental response to NH_3_ exposure in the base gas demonstrates a maximum response at low concentrations and a decrease with increasing concentration values. The observed curve is in accordance with the adsorption theories on zeolites as described in the literature, which are related to the active centers and their storage behavior [63]. In contrast, this sensor principle does not permanently store the analyte, in this case, NH_3_. The sensor signal is the result of a temperature-dependent equilibrium between adsorption and desorption of molecules. This equilibrium explains the concentration-dependent signal.

The selectivity of the sensor is also a consequence of the zeolite’s capability to adsorb specific gas components. In addition to NH_3_ adsorption, water molecules have also been observed to adsorb around the active centers of the zeolite [64,65,66]. An examination of the sensor signal revealed an increase in capacitance values as water content increased from 5 to 10 vol.%.

In comparison, a decrease in the measured sensor signal was observed in the presence of NO (200 ppm). In contrast to the influence of water, this effect was not constant over the entire NH_3_ concentration range. It only had an effect when NO was exposed at the same time to NH_3_. Consequently, it can be assumed that SCR reactions, as depicted in Equation (1), were induced by the low catalytic activity of the zeolite film. This might result in a local reduction in the NH_3_ concentration at the sensor due to the conversion of the analyte, which consequently reduces the output signal. In a previous study (Wöhrl et al. [42]), a correlation between the NO/NO_2_ ratio and temperature was identified through a series of detailed measurements that investigated the impact of NO_x_. The error correlates directly with increasing NO_x_ concentrations, resulting from the higher rate of SCR reactions that convert NH_3_.

**Figure 4:**
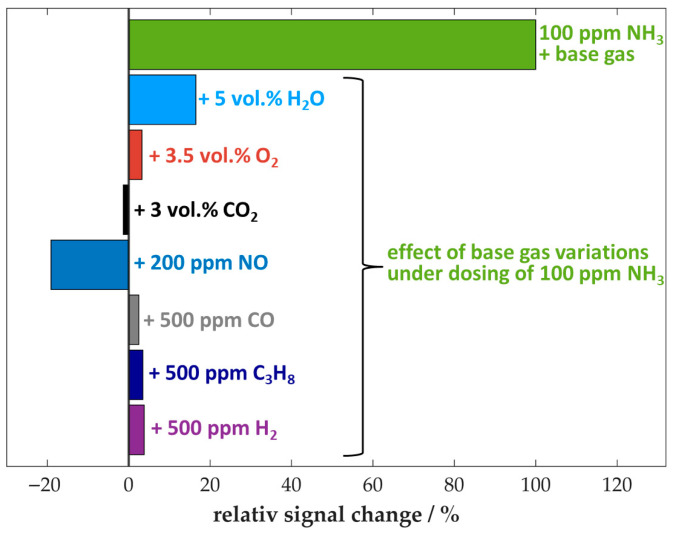
Relative signal change of the sensor when exposed to different gas components in combination with simultaneous dosing of 100 ppm NH_3_, reference value at 100 ppm NH_3_ in the base gas; base gas: 5 vol.% H_2_O, 10 vol.% O_2_, 3 vol.% CO_2_ in N_2_, *T*_sensor_ = 400 °C.

Despite this, the sensor exhibited remarkably low cross-sensitivities to other gas components, including CO and H_2_, which are present in the flue gas of biomass combustion systems. Figure 4 provides a general overview of the sensor’s response when exposed to various gas components under simultaneous dosing of 100 ppm NH_3_.

As previously described, NO_x_ and H_2_O have been identified as the primary causes of cross-sensitivities affecting the sensor. However, the influence of these components is significantly lower than the signal due to NH_3_ exposure. It is important to note that cross-sensitivities are present in the sensor response, with opposite effects. Therefore, it is possible that the two influences may neutralize each other to a certain extent. This is due to the fact that an increase in water content resulting from a more intense combustion is also accompanied by increased NO_x_ emissions due to the formation of fuel-related NO_x_.

The change of the other base gas components (O_2_ and CO_2_) and the dosing of further flue gas components (CO, C_3_H_8_, and H_2_) did not result in any significant changes in the NH_3_ response of the sensor due to the high selectivity of the used zeolite. This can be explained by the fact that no adsorption of these investigated gases occurs at the sensing temperature of 400 °C.

### 3.2. Sensor Poisoning with SO_2_ and HCl

In addition to the cross-sensitivities that have been investigated, which only result in a deviation from the characteristic curve for base gas when present, components that may have a poisoning effect must be considered in biomass combustion. This study focuses on the gases SO_2_ and HCl, which are released during combustion due to the sulfur and chlorine compounds bound in the fuel during the growth process of the plants. As observed with NO_x_ and HC, the concentration ranges for these compounds are significantly influenced by the selected fuel. However, common concentration ranges can be found in the single-digit to low double-digit ppm range.

**Figure 5:**
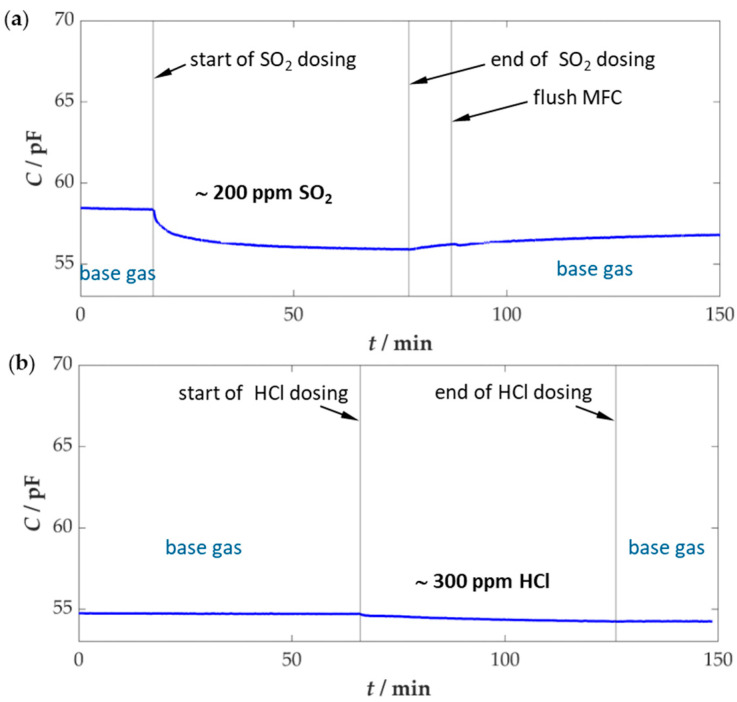
Time course of the test series for sensor poisoning (**a**) with intermediate exposure to approx. 200 ppm SO_2_ and (**b**) with intermediate exposure to about 300 ppm HCl; base gas: ~2 vol.% H_2_O, 10 vol.% O_2_, 3 vol.% CO_2_ in N_2_, *T*_sensor_ = 400 °C.

In order to accelerate any effects of poisoning, the concentration range for SO_2_ was increased to approximately 200 ppm and for HCl to about 300 ppm. As described above, lower concentration ranges could not be achieved with the existing setup, as this would have required extremely low volume flows. This posed a problem for the mass flow controllers used, as it would have compromised the accuracy of the dosing. Consequently, the sensor was exposed to these concentrations for about 1 h, which corresponds to an approximate dose for a duration of about 10 h in real flue gas. Since, as mentioned above, the sensor poisoning took place at a different test setup, where no ammonia could be dosed during this time, a pre- and post-exposure measurement of the sensor was carried out at the actual sensor test bench. The time sequences during the sensor poisoning can be seen in Figure 5.

The sensor poisoning experiments began with a run-in phase of the sensor at base gas, thereby ensuring constant operating conditions. Subsequently, the respective gas was then added for approximately one hour, and the sensor was then operated for a certain time in the base gas again.

**Figure 6:**
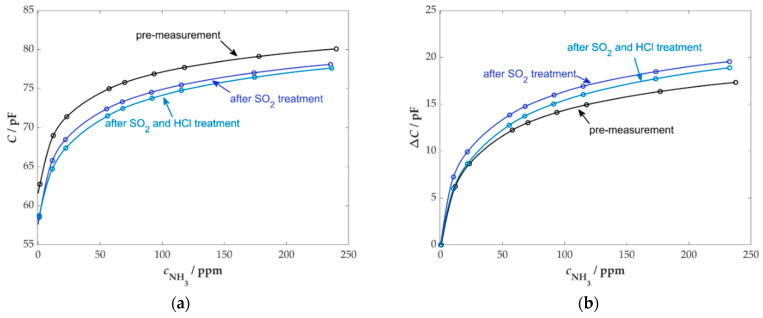
Sensor response in the pre-measurement of the sensor and in the post-measurement after SO_2_ and HCl exposure; (**a**) absolute sensor capacitances; (**b**) capacitance change; base gas: 5 vol.% H_2_O, 10 vol.% O_2_, 3 vol.% CO_2_ in N_2_, *T*_sensor_ = 400 °C.

At the beginning of the SO_2_ exposure, a drop in the sensor capacitance of approximately 2.5 pF was observed, but this stabilized after a short time. After the end of dosing, a slight increase in the signal was again measurable, interrupted by the flushing phase of the SO_2_ MFC, which again led to an increased SO_2_ concentration for a short period. Subsequently, the sensor capacitance increased again, but no return to the initial signal was achieved. A certain ageing effect was also noticeable at the sensor during HCl exposure. At the beginning of dosing, sensor capacitance decreased by approx. 0.5 pF, but this value remained constant even after the end of HCl dosing, and no regeneration effect could be seen. The influence of poisoning gases on the NH_3_ sensitivity of the sensors can be seen in Figure 6.

The post-measurement of the sensor showed that the fundamental behavior changed only slightly due to the exposure to SO_2_ and HCl. Examining the absolute capacitance values (see Figure 6a), we found a reduced base capacitance after SO_2_ and HCl poisoning. This could be attributed to the effects of the removal and subsequent installation of the sensor and the resulting changes in the positioning of the measurement cables. The capacitance change (Δ*C*) in Figure 6b, which is directly related to the initial base capacitance measured at the beginning of the respective experiment, indicates a slight increase in sensor response following SO_2_ exposure. After HCl dosing, the sensor response exhibited a decrease, yet remained above the initial characteristic curve of the sensor. The causes for these effects are not yet fully understood; however, some publications have suggested that SO_2_ may contribute to an increase in the acidity of the active centers in the zeolite [67,68]. This, in turn, results in an enhanced sensor effect. According to the literature, the deactivation of the active centers by HCl could have led to a reduced number of NH_3_ storage sites due to dealumination and, consequently, a decreased sensor response [69,70,71,72].

In general, the ZSM5 zeolite used has a high resistance to poisoning, which also allows it to be used in harsh environmental conditions.

### 3.3. Long-Term Stability

**Figure 7:**
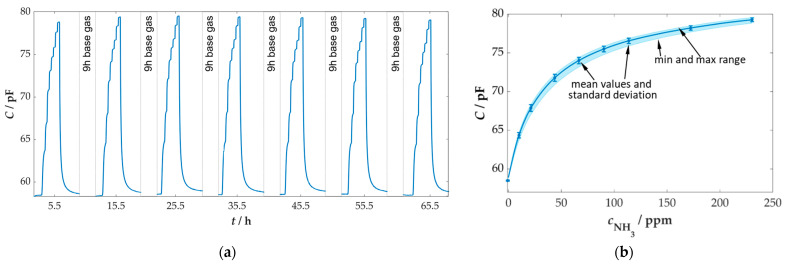
Investigation of the long-term stability of the NH_3_ sensor; (**a**) time course (raw data) of the long-term measurement over almost three days with repeated dosing of ammonia; (**b**) characteristic curve of the sensor with visualization of the standard deviation and the minimum and maximum values; base gas: 5 vol.% H_2_O, 10 vol.% O_2_, 3 vol.% CO_2_ in N_2_, *T*_sensor_ = 400 °C.

The long-term stability of the sensor plays a crucial role in the use of sensors, as repeated recalibrations of sensors would be associated with additional time and costs. The sensor was therefore repeatedly tested for its reaction to ammonia over several days. For this reason, the measurement program shown in Figure 2 was repeated in a 10 h cycle. In between, base gas continued to be dosed, and the sensor was operated at a temperature of 400 °C. The sensor response (raw data) over time is shown in Figure 7a.

The measurement of the long-term stability of the sensor demonstrated a high reproducibility of the sensor effect across a duration of 70 h for this measurement. The baseline signal of the sensor exhibited a negligible variation, maintaining a constant value of 58.5 pF over the duration of the measurement period, which lasted approximately 70 h. Additionally, the response to NH_3_ dosing revealed a high degree of consistency across the total of seven iterations. This finding is further confirmed by the characteristic curve of the sensor, as depicted in Figure 7b, which illustrates the standard deviation for the steady-state values. The maximum and minimum values differ by a maximum of 0.4 pF. This leads to a maximum error in the concentration determination of 3.9%.

### 3.4. Measurement at a Firewood Furnace Under Dynamic Conditions

**Figure 8:**
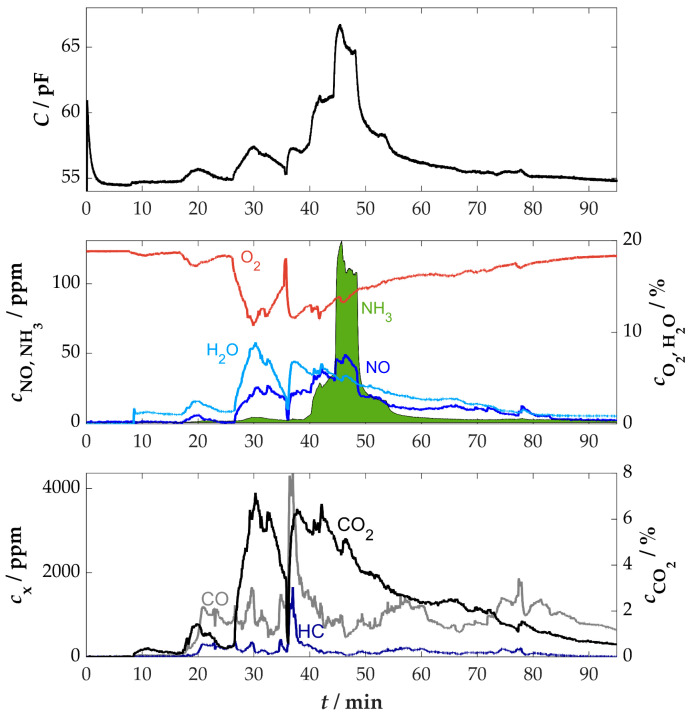
Time course of the sensor signal and the gas concentrations measured by the downstream FTIR spectrometer in the flue gas of a wood-burning stove with ammonia dosing, *T*_sensor_ = 400 °C.

An initial measurement in real flue gas was carried out in the flue pipe of a wood-burning stove for residential use. The purpose of this series of measurements was to further investigate the sensor characteristics in comparison to the laboratory measurements. The manual operation of the stove, in terms of adding firewood or regulating the air supply, creates significantly more dynamic conditions than in laboratory tests. This resulted in high variations of all gas components. According to the findings from the cross-sensitivity measurements in the laboratory, this can influence the sensor signal. While no influence is to be expected from the changes in CO and HC in the range of several thousand ppm, the high variations in water content (1 to 9 vol.%) pose a potential challenge. To better estimate the influences of cross-sensitivities, small amounts of ammonia were added to the intake air, as previously described. This resulted in maximum NH_3_ concentrations of approx. 130 ppm in the flue gas. The time curves of the measurement in combination with the sensor and the downstream FTIR spectrometer can be seen in Figure 8.

The measurement procedure included a short warm-up phase of the sensor, where a stable base value of approximately 54.5 pF was rapidly reached. This value was also reached towards the end of the measurement. The furnace was ignited at approximately *t* = 8 min. During operation, the sensor provided a low-noise signal, with the highest response occurring during the ammonia dosing, from approximately 40 min to 56 min. A direct influence of the high changes in CO_2_, CO, and HC concentration could not be determined. As expected, the increased changes in the water content at the beginning of the measurement (from *t* = 17 to 36 min) resulted in corresponding influences in the sensor signal. Consequently, an increase in the water content might be misinterpreted as an increase in ammonia concentration. Therefore, an approach to correct the sensor signal with the help of a secondary signal is recommended, as a measure to reduce the influence of water on the concentration determination.

**Figure A3:**
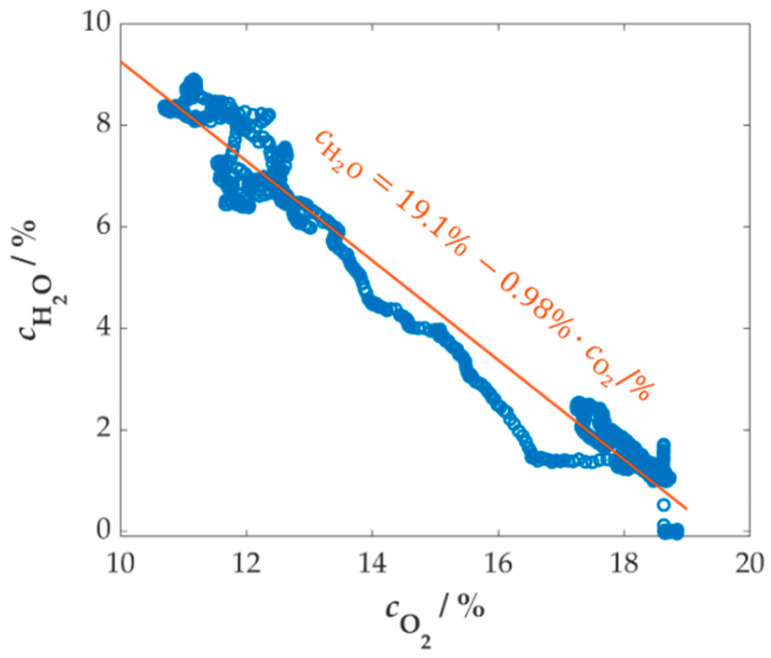
Correlation between the oxygen and water content in the flue gas of the wood-burning furnace (LEDA Unica).

The signal from the lambda probe, which was installed upstream in the flue gas pipe to measure the residual oxygen content, was also continuously monitored. This revealed a certain correlation between the oxygen and water content in the flue gas, which can be explained by the combustion process. As the combustion process intensified, particularly after 26 min, the production of combustion byproducts such as CO_2_ and H_2_O increased. In contrast, the more intense combustion process demands a larger quantity of oxygen, which is reflected in a reduced residual oxygen content in the flue gas [73]. The result of the analysis is an indirectly proportional relationship between the O_2_ and H_2_O content in the flue gas of combustion processes [58]. This relationship is illustrated in Appendix A.2 Figure A3. Based on this linear correlation, the water content can be determined using the signal from the lambda probe, which directly provides the oxygen content in percentage values. The water cross-sensitivity of the sensor can also be corrected using the linear dependencies known from laboratory measurements (≈0.3 pF/vol.%H_2_O).

**Figure 9:**
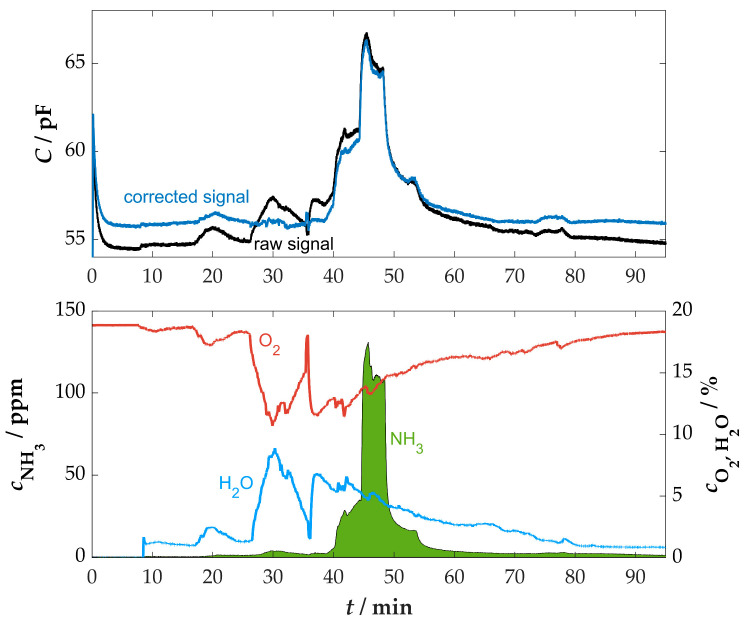
Comparison of the raw signal and the signal of the ammonia sensor corrected by the lambda probe during the measurement on the firewood furnace, *T*_sensor_ = 400 °C.

However, it should be mentioned that this illustrated relationship is not universally valid. The slope and offset of this characteristic curve are subject to variation depending on the type of fuel used or other environmental conditions, like humidity and ambient pressure. Nevertheless, a correction can be made based on a basic knowledge of the correlation, as shown in Figure 9 for this measurement.

**Figure 10:**
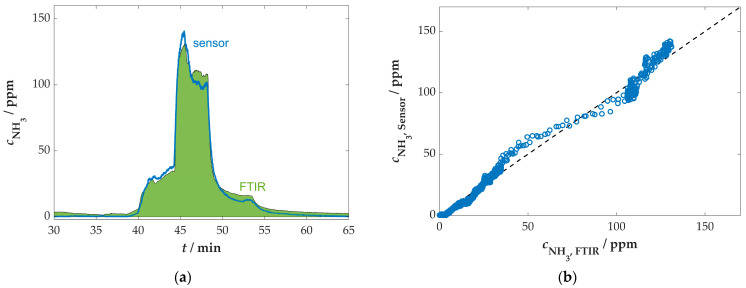
Comparison of the NH_3_ concentration in the flue gas of the wood-burning furnace determined with the sensor (water-corrected) and the FTIR spectrometer: (**a**) time course of the measured data; (**b**) direct comparison of the measured NH_3_ values.

The correction of the sensor signal to a reference value of 5 vol.% H_2_O resulted in a mathematical increase in the sensor signal in areas where the measured/calculated water content is lower than the reference limit value and conversely in areas where the water content is higher than the reference limit value (see Figure 9). Consequently, the fundamental signal of the sensor typically exhibited a higher level, effectively neutralizing the impact of water variations. This recalculated curve subsequently served as the basis for further evaluation. The NH_3_ concentration measured by the sensor was again calculated on the basis of the capacitance change in relation to the base value using the characteristic curve previously determined in the laboratory. As illustrated in Figure 10, the calculated values and the corresponding comparison are presented.

The comparison of the water-corrected response depicted in Figure 10a and the direct comparison illustrated in Figure 10b of the NH_3_ concentration as determined by the sensor and the FTIR spectrometer demonstrates a high degree of agreement. It is evident that minor deviations may have been caused by the time-delayed FTIR measurement due to the piping to the analytic device. These deviations are therefore not necessarily caused by errors in the concentration determination of the FTIR spectrometer or the sensor, but are partly due to the arrangement of the measuring components. In this case, the cross-sensitivity to NO_x_ was negligible due to the minimal NO_x_ emissions of the used stove (less than 100 ppm).

This measurement demonstrated the first successful application of the sensor in the real flue gas of a small-scale combustion plant. The dynamic conditions posed a certain challenge due to the cross-sensitivity of the sensor. However, it was possible to minimize the influence of water in particular by using a secondary signal, in this case, the residual oxygen content determined by a lambda probe.

### 3.5. Measurement on Multi-Fuel Boilers with Straw Pellets Under Stationary Conditions

**Figure 11:**
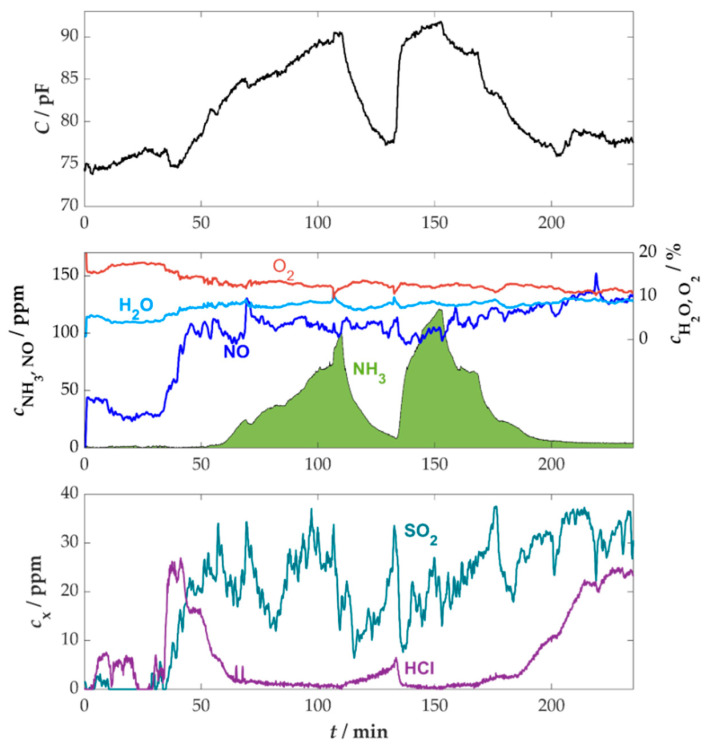
Time course of the sensor signal and the gas concentrations measured by the downstream FTIR spectrometer in the flue gas of a moving grate boiler with straw pellet fuel and AdBlue^TM^ dosing, *T*_sensor_ = 400 °C.

A more realistic field of application was an experiment in the flue gas of a moving grate boiler from REKA, which has a nominal heat output of 120 kW when using straw pellets as fuel (setup in Appendix A.1, Figure A2b). By automatically controlling the air supply using various sensors, such as lambda probes, a stable operating state with a mostly constant flue gas composition can be guaranteed. The raw signals of a measurement with an NH_3_ sensor installed in the flue gas line and an FTIR spectrometer installed downstream of the sensor are shown in Figure 11.

The gas concentrations, as determined by the FTIR spectrometer, confirmed the significantly more stable operation of the system in comparison to the manually controlled wood stove from the previous measurement. The sensor signal exhibited a clear correlation to the NH_3_ concentration occurring in the flue gas, which was dosed with AdBlue^TM^ as previously described. The oxygen and water content maintained relatively stable levels during the measurement period, exhibiting a slight decrease in oxygen and an increase in water. The NO concentration showed an initial rise from approximately 40 ppm to 110 ppm at about 40 min, but subsequently increased only marginally as the experiment progressed. Since the NO concentrations are within a range where the sensor exhibited only minor influences from SCR reactions in the laboratory measurements, these cross-influences can also be neglected in this measurement. Therefore, the impact of fluctuating conditions on the sensor was negligible, and no correction of the sensor signal was needed. Even low concentrations (<40 ppm) of SO_2_ and HCl were detected, which supports the necessity of the previous poisoning investigations.

**Figure 12:**
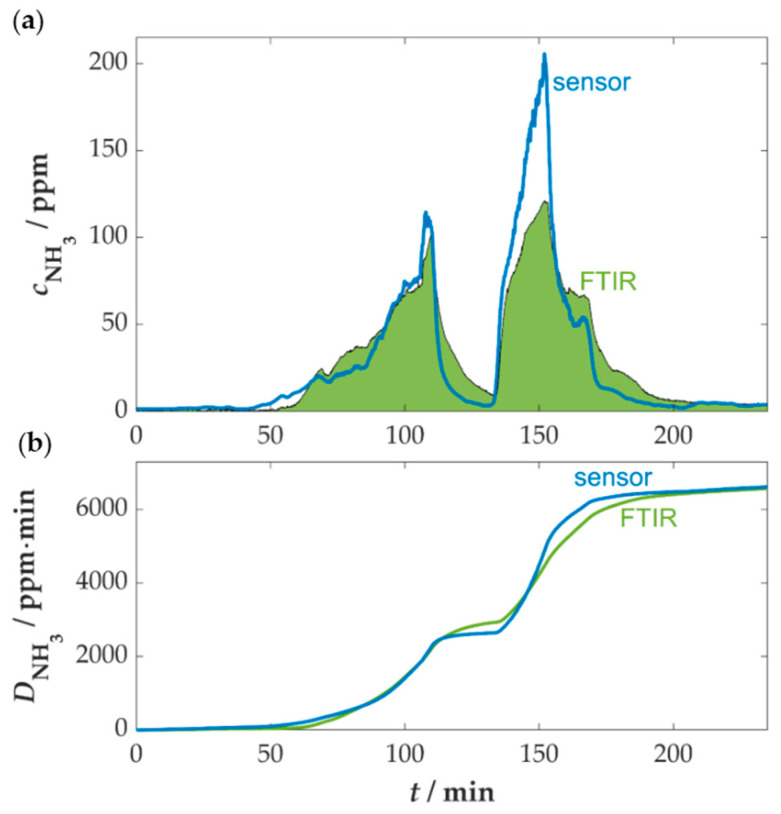
(**a**) Comparison of the time course of the NH_3_ concentration and (**b**) the accumulated NH_3_ dose determined by the sensor and the FTIR spectrometer.

The beginning of the measurement revealed a base capacitance of approximately 74 pF for the sensor, which was higher than the laboratory’s measurement of around 60 pF. This deviation is attributed to the extended length of the measuring cable. This was necessary due to the sensor’s installation at a height of about three meters. Therefore, an increase in the base capacitance was observed for partially unshielded cables. Since the base capacitance of the sensor depends on the position and length of the measuring lines, the sensor needs to be calibrated initially. The base capacitance must be determined at the start of a measurement without dosing ammonia and serves as a base value to be subtracted from the total capacitance for further measurements. Consequently, the change in capacitance, relative to the initial baseline value, was used for subsequent analysis. The sensor can be calibrated either at the start of each measurement or in response to a change in the sensor position. As illustrated in Figure 12, a comparison has been made between the evaluated concentration curve of the sensor and the FTIR.

A direct comparison of the time courses of the NH_3_ concentration measured by the sensor and the FTIR spectrometer revealed initial similarities, despite the presence of differences in phases with rapid concentration changes (see Figure 12a). At the beginning of the measurement, the NH_3_ sensor detected an earlier increase in the NH_3_ concentration (at approximately 50 min) than the FTIR. The decrease in emissions after the AdBlue^TM^ dosage was switched off, which occurs around 115 min after the initial dosage, was also detected earlier by the sensor. Furthermore, the concentration measured at the subsequent increase, approximately 140–150 min later, is considerably higher than that obtained using the FTIR. One possible explanation for the observed discrepancy in concentration measurements could be attributed to the length of the piping between the flue tube and the FTIR measuring cell. Despite the elevated temperatures of the heated pipes, the ammonia reaches the measuring device with a certain latency, which is coupled to the adsorption and desorption processes on the surface of the pipe [74]. This may result in differences to the sensor, which is installed directly in the gas flow and can therefore detect changes in the NH_3_ concentration even faster. In contrast, the sensor exhibits inaccuracies in calculations due to high NH_3_ concentrations (higher than 150 ppm). This is attributed to errors caused by fluctuations in the measurement signal, which are related to the flattening trend of the curve at elevated concentrations.

To ensure a direct comparison of the measurement signals, the total amount of NH_3_ dosed was determined using both measurement systems. This is obtained by calculating the integral of the concentration over time, given by the area under the curve. The comparison is illustrated in Figure 12b. A quantitative analysis reveals that the total amount of NH_3_ as determined by the sensor and the FTIR spectrometer exhibited only minor discrepancies. At the end of the measurement process, the total dose as determined by the sensor was approximately 1.5% lower than that of the FTIR spectrometer. The observed results could be partially attributed to the opposing effects of H_2_O and NO, both of which exhibited a slight increase in concentration during the measurement period. Consequently, the slightly enhanced combustion leads to an elevated fuel conversion, which in turn results in an increased formation of water and fuel NO_x_. The impact of H_2_O on sensor capacitance is known to be positive, while NO has been observed to have a negative effect. However, these opposing influences appear to be balanced to a certain extent, resulting in a largely neutral overall effect.

**Figure A4:**
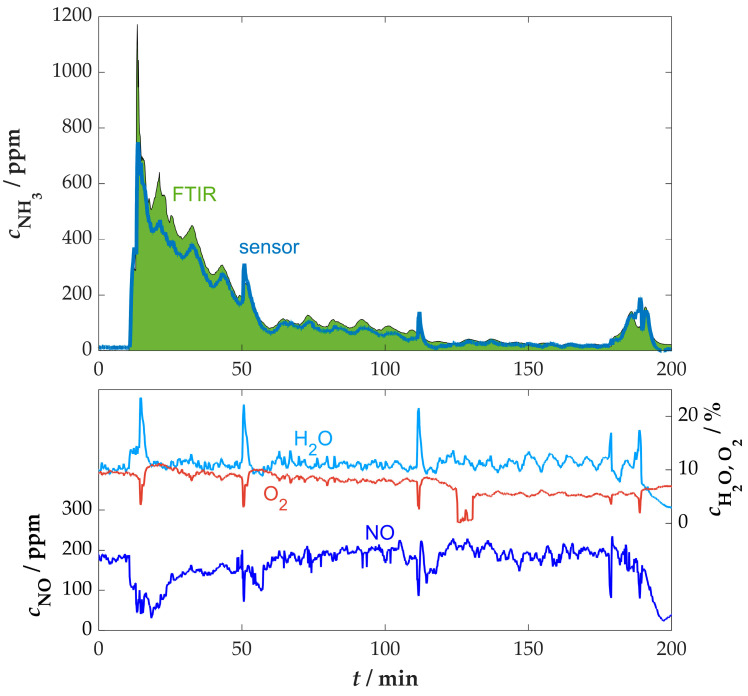
Time profile of the concentration curves of the ammonia sensor compared to the FTIR spectrometer on a multi-fuel boiler (Ökotherm Compact C0), *T*_sensor_ = 400 °C.

**Figure A5:**
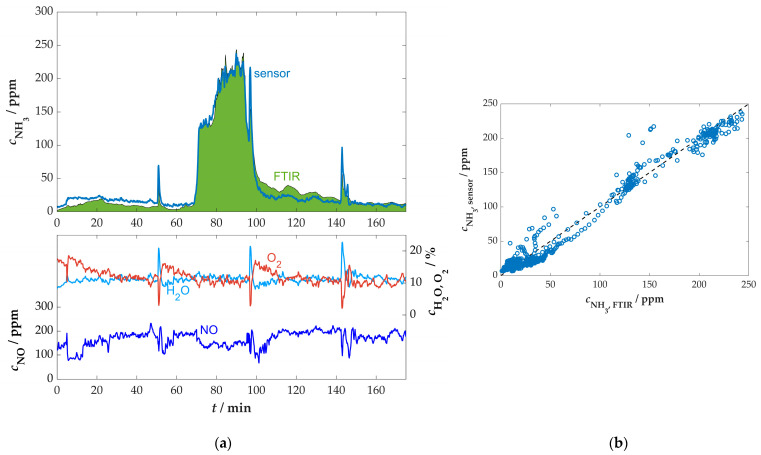
Further measurement in the flue gas of the multi-fuel boiler (Ökotherm Compact C0). (**a**) Comparison of the time curves of the sensor and FTIR; (**b**) comparison of the NH_3_ concentration values.

Further series of measurements with ammonia sensors were carried out at another combustion plant at DBFZ (Ökotherm Compact C0 boiler with ash slider), which was also operated with straw pellets at the nominal heat output of 49 kW. The evaluated sensor measurements with comparison to the FTIR spectrometer can be seen in Figure A4 and Figure A5 in Appendix A.2. The results there were similar to the measurements presented here. Due to the relatively stable conditions in the flue gas of the combustion plant, no correction by secondary signals was necessary, and a high level of agreement was found in concentration ranges <200 ppm. At higher concentrations, however, the deviations were enhanced due to the decreasing sensitivity of the sensor. Previous measurement series showed that sensor-side adjustments to the zeolite composition and operating temperature can achieve higher sensor responses at elevated ammonia concentrations [41,42]. However, this is also linked to a reduced sensitivity at low concentrations or different cross-sensitivities, which would need further investigation. The sensor variant studied here is well-suited for being used as a slip sensor, where mainly concentrations below 100 ppm occur.

## 4. Conclusions and Outlook

The application of the zeolite-based ammonia sensor with capacitive evaluation presented here poses a special challenge in the combustion flue gas of biomass furnaces. The presence of a high number of gas components demands a high degree of selectivity and stability of the sensor. Laboratory tests demonstrated that the cross-sensitivities are generally low; however, the influence of water and nitrogen oxides cannot be fully neglected. Subsequent tests demonstrated high resistivity to SO_2_ and HCl. The long-term stability of the sensor principle showed promising results in the laboratory, but further investigations, especially in real flue gas, are still needed.

The real flue gas measurements could be divided between dynamic and stationary operating conditions. By correcting the water-dependent sensor signal with the help of a lambda probe, a high degree of comparability with the FTIR data could be determined in the dynamic application case on a firewood furnace. In the stationary operation of multi-fuel boilers, no correction of the cross-influences was necessary. In these experiments, the ammonia sensor also showed a high correlation with the FTIR spectrometer, although there were minor deviations in the comparison of the time curves due to latencies caused by the piping. The sensor also lost accuracy at high concentration values (>200 ppm NH_3_) due to the non-linear characteristic curve.

This sensor principle is therefore suitable for slip detection, with the purpose of recognizing a breakthrough or an overdosage of AdBlue^TM^. In the context of legal requirements, in which NH_3_ slip limits in certain applications are set below 50 ppm, the sensor showed the highest response to ammonia within low-concentration ranges of less than 100 ppm. Therefore, a further series of measurements for NH_3_ slip detection in real exhaust gas with SCR systems, including automotive applications, is planned for the future. The sensor is to be used there to detect NH_3_ emissions subsequent to the SCR catalytic converter. This will also entail more intensive investigations into reproducibility and sensor aging caused by the exhaust gas. Particular emphasis will be placed on the long-term stability of the sensor, where measurement series will be carried out over an extended period of time in real flue gas.

## Data Availability

The raw data supporting the conclusions of this article will be made available by the authors on request.

## Abbreviations

The following abbreviations are used in this manuscript:

BImschV	Verordnung zur Durchführung des Bundes-Immisionsschutzgesetzes
SCR	Selective catalytic reduction
MOS	Metal-oxide semiconductor
NDIR	Non-dispersive infrared spectroscopy
SAW	Surface acoustic wave
FTIR	Fourier-transform infrared spectroscopy
ZSM5	Zeolite Socony Mobil-5
MFI	Metal framework inverted
SEM	Scanning electron microscope
IDE	Interdigital electrode
DBFZ	Deutsches Biomasseforschungszentrum
MFC	Mass flow controller
